# The Virtual Feedback Loop: Psychometric Validation of a New Scale to Measure Digital Validation Seeking in Higher Education

**DOI:** 10.3390/ejihpe16030032

**Published:** 2026-02-27

**Authors:** Mohamed Ali Nemt-allah, Mamdouh Mahmoud Mostafa, Mamdouh Mosaad Helali, Hussam Khalifah Aldawsari, Bandar Saud Alromaih, Ashraf Ragab Ibrahim

**Affiliations:** 1 Educational Psychology and Statistics Department, Faculty of Education, Al-Azhar University, Cairo 35822, Egypt; ashrafibrahem.26@azhar.edu.eg; 2 Mental Health Department, Faculty of Education, Al-Azhar University, Cairo 11765, Egypt; mamdouhbadawy.197@azhar.edu.eg; 3 The National Research Center for Giftedness and Creativity, King Faisal University, Al-Ahsa 31982, Saudi Arabia; 4 Psychology and Education Department, College of Education, King Faisal University, Al-Ahsa 31982, Saudi Arabia; haldawsari@kfu.edu.sa; 5 Educational Leadership Department, College of Education, King Faisal University, Al-Ahsa 31982, Saudi Arabia; balrumaih@kfu.edu.sa

**Keywords:** digital validation seeking, psychometric validation, higher education, social media, scale development, student well-being

## Abstract

Despite the pervasive role of digital platforms in contemporary higher education, existing measurement tools fail to capture students’ psychological dependence on online approval within academic contexts, focusing instead on technical competencies or clinical addiction symptoms. This study developed and psychometrically validated the Digital Validation Seeking Scale (DVSS), a multidimensional instrument measuring university students’ reliance on digital feedback for academic and identity confirmation. Two independent samples of Egyptian undergraduate students were recruited: an exploratory sample of 511 students and a confirmatory sample of 740 students from six universities. The DVSS underwent rigorous content validation by eleven experts, exploratory factor analysis (EFA) using Principal Axis Factoring with Promax rotation, and confirmatory factor analysis (CFA) comparing competing structural models. Results revealed a robust four-factor structure comprising Academic Self-Quantification (ASQ), Feedback Hyper-vigilance (FHV), Social Comparison (SC), and Performative Studiousness (PS), with the first-order four-factor model demonstrating superior fit indices. The final 19-item scale exhibited excellent internal consistency, with Cronbach’s alpha coefficients ranging from 0.807 to 0.938 for subscales and total score, respectively, and strong test–retest reliability. The DVSS provides researchers and practitioners with a theoretically grounded, psychometrically sound instrument for identifying maladaptive digital validation patterns before they compromise academic engagement or psychological well-being, enabling targeted interventions within hybrid educational environments.

## 1. Introduction

In contemporary higher education, students increasingly turn to digital platforms not merely for information access but to obtain external confirmation of their competence, identity, and belonging. Digital validation seeking can be defined as a recurrent, intentional pattern of using digital platforms and tools to obtain social, academic, or identity-related confirmation from others or from systems, such that external digital feedback becomes a key regulator of students’ self-evaluation and academic decisions (X. Zhang et al., 2025). This phenomenon manifests through multiple channels: students seek identity validation by posting personal narratives in online forums to confirm self-suspected conditions (X. Zhang et al., 2025); pursue academic competence validation by consulting AI tools and learning analytics to reassure themselves about understanding (Cabero-Almenara et al., 2022; Muñoz-Repiso et al., 2020; Pumptow & Brahm, 2020); engage in SC through monitoring peers’ digital achievements (Chen et al., 2025; Neagu & Vieriu, 2025); and seek health-related normality validation during crises (Htay et al., 2022; Neagu & Vieriu, 2025).

Despite the proliferation of social media measurement tools, existing scales inadequately capture digital validation seeking within university contexts. Current instruments predominantly focus on problematic use and addiction symptoms (Boer et al., 2021; Eijnden et al., 2016; Fung, 2019; Sadeghi et al., 2025), which emphasize clinical impairment rather than the everyday micro-processes of seeking likes, comments, and peer approval that characterize student digital behavior. Adjacent constructs such as digital media self-efficacy (Pumptow & Brahm, 2020), digital competence (Tzafilkou et al., 2022), and digital life balance (Lima-Costa et al., 2024; Malas et al., 2025) measure skills, confidence, or overall online-offline equilibrium but fail to probe students’ emotional dependence on peer approval and validation metrics. Furthermore, scales targeting social media consciousness (Choukas-Bradley et al., 2020), fatigue (S. Zhang et al., 2021), and anxiety (Alkis et al., 2017) were designed for different populations or contexts, missing university-specific validation dynamics around academic identity, performance, and institutional belonging that define contemporary student experience.

University students, particularly those aged 18–24, represent the primary stakeholders affected by digital validation loops, with significant implications for both academic and psychological outcomes (Atlam et al., 2021; Elsayed, 2025; Wang et al., 2021). Research demonstrates that digital engagement patterns influence academic performance through complex pathways: while some studies report positive associations between social media use and grade point average (GPA) (Elsayed, 2025; Shahzad et al., 2024), excessive reliance on digital validation can undermine achievement when it substitutes meaningful human support (Crawford et al., 2024). The psychological toll is equally concerning, as students experience social network exhaustion from constant comparison (Pang & Zhang, 2024), academic burnout from cognitive overload (Wang et al., 2021), and diminished well-being when digital interactions replace authentic connections (Crawford et al., 2024; Neagu & Vieriu, 2025). Female students and those from lower socioeconomic backgrounds appear particularly vulnerable to digital stressors (Wang et al., 2021), highlighting the need for targeted measurement tools that capture these nuanced experiences.

The contemporary academic environment exists as a hybrid assemblage where physical classrooms intersect with learning management systems, social media platforms, and messaging applications, creating entangled spaces where validation seeking fundamentally shapes learning outcomes (Hashim et al., 2021; Lamb et al., 2021; Tondeur et al., 2024). Within these integrated environments, social media used for collaborative learning enhances peer interaction, knowledge sharing, and academic performance when appropriately channeled (Al-Rahmi et al., 2022; Ansari & Khan, 2020). Digital communication tools that foster connectedness, immediacy, and teacher presence transform validation needs into productive engagement through prompt feedback and visible participation (El-Sayad et al., 2021; Hehir et al., 2021; Raes, 2021). However, unbounded digital exposure can redirect validation seeking toward status comparison and non-academic social activities, potentially undermining professionalism and well-being (Guckian et al., 2021). Hybrid courses deliberately balancing technology, pedagogy, and social interaction demonstrate superior academic performance and satisfaction (Abdigapbarova et al., 2025; Mahender & Sharma, 2025), suggesting that how hybrid spaces channel validation drives—toward collaboration or fragmented attention—critically determines educational outcomes.

While digital validation seeking is nearly universal among students, it becomes maladaptive when characterized by compulsive pursuit of online approval as a central regulator of self-worth (Nesi & Prinstein, 2018; X. Zhang et al., 2025). Research identifies critical warning markers: effortful digital status seeking prospectively predicts substance use and sexual risk behaviors (Nesi & Prinstein, 2018), while approval-seeking schemas underpin behavioral addictions and negative self-image (Ang et al., 2025; Vieira et al., 2023). Among university students, psychological distress and online academic difficulties co-occur, negatively affecting course satisfaction and performance (Curelaru & Curelaru, 2024), yet students struggle to discern trustworthy digital mental health resources despite high need (Montagni et al., 2020). The urgency of early measurement is underscored by evidence that unmanaged digital threats can escalate to burnout and mental health distress (Sun et al., 2022), and that digital interventions can effectively support well-being when appropriately targeted (Ferrari et al., 2022; Lattie et al., 2019). Measuring validation-seeking patterns before they compromise academic engagement or clinical thresholds is therefore essential for institutional prevention efforts.

The virtual feedback loop operates through two complementary theoretical mechanisms: social comparison theory and self-determination theory (SDT). From a social comparison perspective, algorithmically curated digital metrics provide continuous standards against which students gauge their relative standing, with comparison orientation mediating how feedback impacts state self-esteem—amplifying both benefits of positive feedback and harms of negative feedback (Chen, 2025; García-Arch et al., 2025). Through an SDT lens, quantified feedback dynamically regulates basic psychological needs: likes and comments function as competence-relevant information, digital interactions signal relatedness satisfaction or thwarting, and platform affordances either support autonomous engagement or promote controlled, contingent behavior driven by external validation (Gagné et al., 2022; Ryan et al., 2018, 2021; Urhahne & Wijnia, 2023; Q. Zhang & Shakibaei, 2025). This recursive cycle—where digital action elicits feedback that shapes self-evaluation and subsequent behavior—creates a self-perpetuating loop wherein comparison processes and need regulation mutually reinforce students’ dependence on external digital validation.

Current educational measurement tools overwhelmingly assess technical and functional digital competencies—device proficiency, information search, content creation, safety, and ethics—derived from frameworks like DigComp and DigCompEdu (Cabero-Almenara et al., 2020; Fan & Wang, 2022; Nguyen & Habók, 2023; Tzafilkou et al., 2022), yet fail to capture the psychological construct of digital validation seeking within academic contexts. Systematic reviews consistently identify critical gaps: limited availability of validated, context-sensitive instruments (Suri et al., 2025), absence of authentic performance assessment (Suri et al., 2025), and neglect of socio-emotional dimensions such as dependence on online approval and feedback-seeking motives in educational platforms (Nguyen & Habók, 2023; Suri et al., 2025). Recent role-specific scales for teachers, students, and parents continue to operationalize what learners can do rather than why and how they seek validation online (Jia et al., 2025; Pelaez-Sanchez et al., 2024; Soriano-Alcantara et al., 2024; Viberg et al., 2024). This study addresses this void by developing the first psychometrically validated, education-specific instrument measuring digital validation seeking in higher education.

Given the proliferation of digital platforms in higher education and the absence of validated instruments measuring students’ psychological dependence on online approval within academic contexts, this study aimed to develop and psychometrically validate the Digital Validation Seeking Scale (DVSS) for university students. Specifically, the research sought to generate a theoretically grounded item pool capturing multiple dimensions of digital validation seeking in educational settings; establish content validity through expert review procedures; determine the underlying factor structure through EFA with an independent sample; confirm the optimal factorial model using CFA; assess internal consistency reliability using multiple coefficients; evaluate temporal stability through test–retest procedures; and examine convergent and discriminant validity through interfactor correlations and composite reliability (CR) indices. By providing a psychometrically sound measurement tool specifically designed for higher education contexts, this study addresses critical gaps identified in systematic reviews and enables future research examining relationships between digital validation seeking and academic outcomes, psychological well-being, and educational intervention effectiveness.

## 2. Materials and Methods

### 2.1. Participants and Sampling

The present study adopted a cross-sectional design utilizing two independent samples of Egyptian undergraduate students to conduct exploratory and confirmatory factor analyses sequentially. The exploratory sample comprised 511 students, whereas the confirmatory sample consisted of 740 students, drawn from six geographically distributed Egyptian universities: Al-Azhar University, Zagazig University, Kafr El Sheikh University, Beni Suef University, Damanhour University, and Tanta University. The complete demographic characteristics of both samples are presented in Table 1. Both samples demonstrated relatively balanced gender distributions, with males constituting approximately 53.0% and 52.6% of the exploratory and confirmatory samples, respectively.

Representation across all four academic years was proportionally equitable, with year-level distributions ranging from 23.3% to 27.4% in the exploratory sample and from 24.5% to 25.4% in the confirmatory sample. Urban residence was marginally more prevalent than rural residence across both samples. Participants in the exploratory sample ranged in age from 18 to 24 years (M = 21.18, SD = 2.03), while those in the confirmatory sample ranged from 18 to 23 years (M = 20.47, SD = 1.70). To formally verify age equivalence, an independent-samples t-test was conducted, confirming no statistically meaningful difference between the two groups and thereby supporting sample comparability.

To confirm broader demographic equivalence, chi-square homogeneity tests revealed no statistically significant differences in gender (χ^2^ = 0.021, df = 1, *p* = 0.885), academic year (χ^2^ = 0.412, df = 3, *p* = 0.938), or residence (χ^2^ = 1.124, df = 1, *p* = 0.289), collectively indicating that the two samples were demographically homogeneous and unlikely to introduce differential sampling bias into the factor analytic procedures. With respect to digital platform usage, participants reported engagement across multiple social media platforms, including Facebook, TikTok, Twitter, Snapchat, and Instagram, with Facebook emerging as the most frequently reported platform within the confirmatory sample.

### 2.2. Procedure

Data collection occurred between 10 and 25 November 2025, using Google Forms to distribute the survey instruments electronically. This online administration method ensured broad accessibility across the geographically dispersed university locations and facilitated efficient data collection during the academic semester. For test–retest reliability assessment, a subsample of 171 students completed the scale on two occasions separated by a 15-day interval, a duration sufficient to minimize memory effects while remaining brief enough to ensure stability of the construct being measured.

### 2.3. Instrument Development

The Digital Validation Seeking Scale (DVSS) was developed to measure the extent to which university students seek external confirmation and approval through digital platforms in academic contexts. The initial item pool consisted of 24 statements, consistent with recommendations that item pools should contain approximately two to three times the intended final scale length to allow for attrition during validation (DeVellis, 2016; Boateng et al., 2018). These items were designed to capture various dimensions of digital validation seeking behaviors, including dependence on social media feedback for academic self-evaluation, compulsive monitoring of engagement metrics, social comparison with peers’ digital achievements, and performative presentation of study behaviors.

All items were rated on a five-point Likert scale ranging from 1 (strongly disagree) to 5 (strongly agree), a format widely recommended for attitudinal measurement in educational and psychological research due to its balance between response sensitivity and cognitive manageability (Likert, 1932; Revilla et al., 2014; Wakita et al., 2012), with higher scores indicating greater levels of digital validation seeking, following standard unidirectional scoring conventions for behavioral tendency scales (DeVellis, 2016).

### 2.4. Content Validity

The initial 24-item version of the DVSS underwent rigorous expert review to establish content validity. Eleven experts specializing in educational psychology, mental health, and educational technology were recruited, consistent with recommendations that content validity panels comprise between five and fifteen subject matter experts to ensure stable CVI estimates (Lynn, 1986; Polit & Beck, 2006). These experts evaluated each item for appropriateness of response alternatives, clarity of wording, and alignment with the scoring key. Expert ratings were quantified to calculate the Content Validity Index (CVI), which reached 0.803, exceeding the conventional threshold of 0.80 for acceptable content validity recommended for multi-item scales (Polit et al., 2007; Lynn, 1986). Based on expert feedback and quantitative analysis of item-level agreement, four items were eliminated following established item-level CVI (I-CVI) criteria whereby items rated appropriate by fewer than 78% of experts are recommended for removal (Polit et al., 2007; Almanasreh et al., 2019), resulting in a refined 20-item instrument that proceeded to psychometric evaluation through EFA.

### 2.5. Data Analysis

All statistical analyses were conducted using SPSS version 27 for EFA and reliability assessment, and AMOS version 26 for CFA and structural equation modeling. The EFA employed Principal Axis Factoring as the extraction method, chosen following formal assessment of distributional assumptions using the Kolmogorov–Smirnov and Shapiro–Wilk goodness-of-fit tests (Razali & Wah, 2011), which indicated statistically significant departures from normality for all 20 items. Principal Axis Factoring is recommended over Maximum Likelihood extraction under such conditions due to its robustness when multivariate normality cannot be confirmed (Fabrigar et al., 1999; Floyd & Widaman, 1995), with Promax rotation to allow for correlated factors. Parallel analysis was conducted to determine the optimal number of factors to retain by comparing eigenvalues from the actual data with those from random data. Model fit in CFA was evaluated using multiple indices including the chi-square statistic, Comparative Fit Index (CFI), Root Mean Square Error of Approximation (RMSEA), and standardized residuals. Reliability was assessed using Cronbach’s alpha, McDonald’s omega, and Guttman’s lambda-2. Internal consistency was examined through inter-factor and item-total correlations, and temporal stability was evaluated through test–retest correlations using Pearson’s r.

## 3. Results

Before conducting factor analysis, preliminary analyses were performed to ensure the data met necessary assumptions and that the sample was adequate for the planned analyses. The descriptive statistics revealed mean scores ranging from 2.29 to 3.10, with standard deviations ranging from 1.26 to 1.41, indicating reasonable variability across items and the absence of ceiling or floor effects. To formally assess the normality of the data distribution, Kolmogorov–Smirnov and Shapiro–Wilk goodness-of-fit tests were conducted for all 20 items using SPSS version 27. Results indicated statistically significant deviations from normality across all items (K-S statistics ranging from 0.154 to 0.218, *p* < 0.001; S-W statistics ranging from 0.847 to 0.893, *p* < 0.001), as presented in Table 2. These findings are consistent with the observed skewness values ranging from −0.023 to 0.693 and kurtosis values ranging from −1.240 to −0.646, and collectively confirm that strict normality assumptions were not met. Accordingly, Principal Axis Factoring was selected as the extraction method for EFA due to its robustness under non-normal distributions (Fabrigar et al., 1999; Floyd & Widaman, 1995), and the use of Promax oblique rotation was retained as planned.

The Kaiser-Meyer-Olkin (KMO) measure of sampling adequacy yielded a value of 0.946, substantially exceeding the recommended minimum of 0.60 and indicating that the correlation patterns among items were appropriate for factor analysis. Bartlett’s Test of Sphericity was highly significant (χ^2^ = 5856.148, df = 190, *p* < 0.001), confirming that correlations among items were sufficiently large to warrant factor extraction. These indices collectively demonstrated that the data were suitable for EFA.

To determine the optimal number of factors to extract, both traditional eigenvalue criteria and parallel analysis were employed. The initial extraction using Principal Axis Factoring revealed that the first factor accounted for 47.45% of the total variance with an eigenvalue of 9.490, substantially larger than subsequent factors. Table 3 presents the total variance explained by each potential factor in both the initial extraction and after applying extraction criteria.

**Figure 1:**
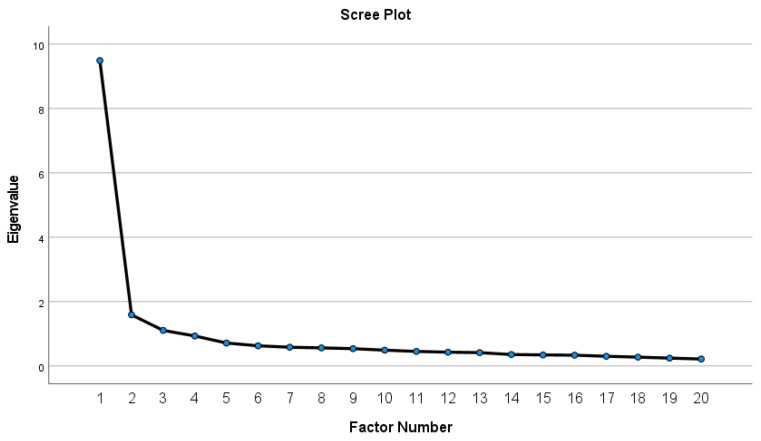
Scree Plot for EFA.

The scree plot (Figure 1) showed a clear elbow after the first factor, with subsequent factors showing much smaller eigenvalues. However, three additional factors demonstrated eigenvalues slightly above 1.0.

To adjudicate between unidimensional and multidimensional solutions, parallel analysis was conducted comparing observed eigenvalues against those generated from random data with equivalent dimensions. Table 4 presents the comparison between real data eigenvalues and simulated data eigenvalues.

The parallel analysis results indicated that four factors should be retained, as the first four observed eigenvalues (10.140, 1.084, 0.595, and 0.411) exceeded their corresponding random data eigenvalues (0.455, 0.319, 0.266, and 0.219, respectively). Factor 5’s observed eigenvalue (0.111) fell below the random threshold (0.189), clearly indicating that extraction should cease after four factors. Based on these convergent indicators, a four-factor solution was pursued for further analysis. The four-factor solution using Principal Axis Factoring with Promax rotation converged in six iterations and extracted 57.29% of the total variance. Table 5 presents the pattern matrix showing item loadings on each of the four extracted factors.

The pattern matrix revealed a theoretically interpretable structure with items loading cleanly on their respective factors. Factor 1, labeled FHV, comprised five items (6, 7, 8, 9, 10) with pattern coefficients ranging from 0.664 to 0.901, reflecting compulsive monitoring of digital responses and anxiety about delayed feedback. Factor 2, labeled PS, included five items (16, 17, 18, 19, 20) with loadings between 0.611 and 0.793, capturing staged presentations of academic effort for digital audiences. Factor 3, labeled Social Comparison (SC), consisted of five items (11, 12, 13, 14, 15) with coefficients from 0.571 to 0.854, measuring competitive tracking of peers’ digital achievements. Factor 4, labeled ASQ, contained four items (1, 2, 3, 4) with loadings ranging from 0.506 to 0.809, representing reliance on engagement metrics to evaluate academic competence. One item (Item5) failed to load substantially on any factor and showed the highest uniqueness value (0.581), suggesting weak communality with the underlying factor structure.

Communalities for the four-factor solution ranged from 0.354 to 0.723, indicating that the extracted factors accounted for adequate proportions of item variance. The factor correlation matrix (Table 6) revealed moderate to strong positive intercorrelations among all four factors, supporting the use of oblique rotation and suggesting that these dimensions represent related facets of an overarching digital validation seeking construct.

**Figure 2:**
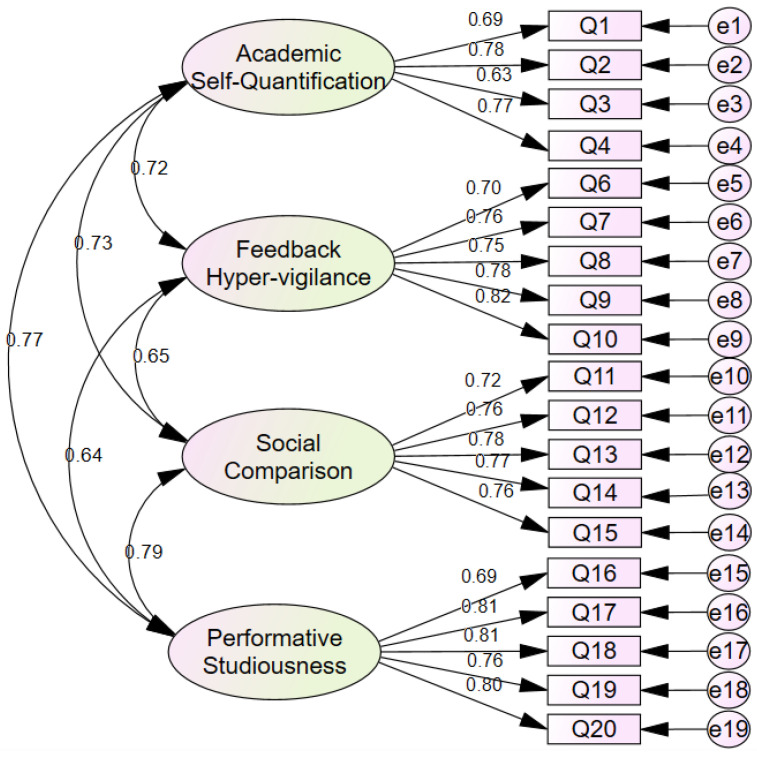
First-Order Four-Factor Model with Standardized Parameter Estimates.

These substantial interfactor correlations (ranging from 0.620 to 0.744) provided empirical justification for testing higher-order factor models in subsequent confirmatory analyses. Three competing structural models were evaluated using the confirmatory sample to determine the optimal representation of the DVSS factor structure: a unidimensional model wherein all 20 items loaded on a single general factor, a first-order multidimensional model with four correlated factors, and a second-order model with four first-order factors loading on a single higher-order Digital Validation Seeking factor. Figure 2 presents the path diagram for the first-order four-factor model, which demonstrated the best fit.

Table 7 presents the comparative fit indices for these three models, facilitating direct evaluation of their relative adequacy.

The unidimensional model demonstrated poor fit across all indices, with an elevated RMSEA of 0.110 (well above the acceptable threshold of 0.08), inadequate CFI of 0.812 (below the recommended 0.90 minimum), and an excessively large chi-square to degrees of freedom ratio of 9.963. These results decisively rejected the hypothesis that digital validation seeking could be adequately represented as a single undifferentiated construct. In contrast, the first-order four-factor model exhibited excellent fit to the data. The RMSEA of 0.046 fell well within the acceptable range, indicating close approximate fit. The CFI of 0.970 and NFI of 0.952 substantially exceeded conventional benchmarks, demonstrating that the model accounted for the vast majority of observed covariances. The normed chi-square ratio of 2.594 was well within acceptable limits, and both GFI (0.949) and AGFI (0.934) indicated strong absolute and parsimony-adjusted fit. The low RMR value of 0.053 further confirmed excellent model fit.

The second-order model, which imposed an additional hierarchical structure with the four first-order factors loading on a higher-order Digital Validation Seeking factor, also demonstrated good fit with an RMSEA of 0.048, CFI of 0.968, and NFI of 0.950. However, comparison of the first-order and second-order models revealed that the more parsimonious first-order structure achieved slightly superior fit with two fewer degrees of freedom, as evidenced by marginally better CFI (0.970 vs. 0.968) and RMSEA (0.046 vs. 0.048) values. The chi-square difference between models was minimal (Δχ^2^ = 18.005, Δdf = 2), and the more constrained second-order model did not offer meaningful improvement in interpretability or theoretical coherence.

Based on the principle of parsimony and the superior fit indices, the first-order four-factor model was retained as the optimal representation of the DVSS structure for the purposes of scale validation and subscale-level score interpretation. It is acknowledged, however, that when the DVSS total construct is employed alongside other constructs within larger structural equation models, the second-order representation may offer practical advantages by consolidating the four dimensions into a single higher-order factor, thereby reducing the number of structural paths and simplifying model estimation (Hair et al., 2019; Marsh et al., 2020). Researchers are therefore encouraged to select the representation most appropriate to their specific research objectives.

Reliability analyses were conducted separately for each of the four dimensions and for the total scale using the confirmatory sample. Table 8 presents the comprehensive reliability coefficients for each subscale and the overall instrument.

All four subscales demonstrated excellent internal consistency, with Cronbach’s alpha coefficients ranging from 0.807 to 0.882, substantially exceeding the conventional threshold of 0.70 for acceptable reliability. McDonald’s omega coefficients, which are less influenced by the number of items and do not assume tau-equivalence, showed nearly identical values ranging from 0.810 to 0.883, confirming the stability of reliability estimates across different computational approaches. Guttman’s lambda-2 coefficients, representing the greatest lower bound of reliability, were virtually identical to alpha and omega values, further corroborating the precision of these estimates. The total scale demonstrated outstanding reliability, with all three coefficients reaching 0.938 or 0.939, indicating that the full 20-item instrument (excluding Q5) produces highly consistent measurements of digital validation seeking.

For the first-order four-factor model, CR and AVE were calculated to assess convergent validity. ASQ achieved a CR of 0.811 and AVE of 0.519, while FHV, SC, and PS dimensions showed CR values of 0.874, 0.871, and 0.883, respectively, with AVE values of 0.582, 0.574, and 0.602. These values indicated that the extracted factors accounted for substantial proportions of item variance and that convergent validity was established, as CR values exceeded 0.70 and AVE values approached or exceeded the 0.50 benchmark. To assess the discriminant validity and internal structure of the scale, correlations among the four subscales and with the total score were examined. Table 9 displays these intercorrelations for the confirmatory sample.

All subscale intercorrelations were positive, statistically significant at the 0.01 level, and ranged from 0.570 to 0.697, indicating that the four dimensions were moderately to strongly related yet sufficiently distinct to warrant separate measurement. These correlations support the conceptualization of digital validation seeking as a multifaceted construct with interrelated but distinguishable behavioral manifestations. Each subscale demonstrated very strong correlations with the total score (r = 0.820 to 0.870), confirming that all four dimensions contributed substantially to the overarching construct while retaining their unique variance.

Test–retest reliability was assessed using a subsample of 171 students who completed the DVSS on two occasions separated by a 15-day interval. Pearson correlation coefficients between Time 1 and Time 2 scores were calculated for each subscale and the total score. ASQ demonstrated a correlation of 0.546 (*p* < 0.01), FHV showed r = 0.691 (*p* < 0.01), SC yielded r = 0.782 (*p* < 0.01), and PS achieved r = 0.791 (*p* < 0.01). The total scale test–retest correlation was 0.830 (*p* < 0.01), indicating strong temporal stability. These coefficients confirm that the DVSS produces consistent measurements across time, with the total score demonstrating particularly robust stability. The somewhat lower correlation for ASQ, while still acceptable, suggests that this dimension may be more situationally variable or sensitive to recent academic experiences than the other three dimensions.

## 4. Discussion

The present study successfully developed and validated the DVSS, a 19-item instrument comprising four distinct yet interrelated dimensions: ASQ, FHV, SC, and PS (see Appendix A). The first-order four-factor model demonstrated superior fit compared to unidimensional and second-order alternatives, suggesting that digital validation seeking manifests through multiple behavioral pathways rather than a single undifferentiated tendency. The strong interfactor correlations (0.570–0.697) indicate these dimensions represent related facets of an overarching construct, consistent with theoretical frameworks positing that students engage validation-seeking behaviors across multiple digital contexts. Excellent internal consistency (α = 0.807–0.882) and robust test–retest stability (r = 0.830 for total scale) confirm the DVSS produces reliable measurements, while content validity procedures and convergent validity indices establish its construct appropriateness for capturing university students’ reliance on digital platforms for academic and identity confirmation.

It is important to acknowledge that a second-order formative conceptualization of digital validation seeking was considered as a structural alternative. In a formative model, the four dimensions would be treated as causally antecedent components that collectively define the higher-order construct, rather than as reflective manifestations of it (Diamantopoulos & Winklhofer, 2001; MacKenzie et al., 2005). While each dimension captures a qualitatively distinct behavioral domain, three considerations favored the reflective specification. First, the substantial intercorrelations among dimensions (r = 0.570–0.697) are more consistent with reflective than formative logic, which typically assumes component independence (Jarvis et al., 2003). Second, items within each subscale were constructed as interchangeable indicators of a common underlying tendency. Third, AVE values exceeding 0.50 and CR values exceeding 0.70 support the reflective interpretation (Fornell & Larcker, 1981).

Nonetheless, it is important to note that the choice between first-order and second-order representations carries practical implications beyond model fit. When the DVSS is incorporated into broader structural models alongside other constructs, the second-order specification reduces model complexity by replacing four separate first-order relationships with a single path from the higher-order Digital Validation Seeking factor, which may improve model parsimony and estimation stability in such contexts (Hair et al., 2019; Marsh et al., 2020). The good fit of the second-order model (RMSEA = 0.048, CFI = 0.968) confirms that this representation remains psychometrically viable and is recommended for use in future research employing the DVSS within multi-construct structural frameworks.

These findings extend existing research by addressing critical measurement gaps identified in systematic reviews regarding socio-emotional dimensions of digital behavior in educational contexts. While previous instruments focused on technical competencies and problematic use symptoms, the DVSS uniquely captures the everyday micro-processes through which students seek peer approval and external confirmation that characterize contemporary academic life. The moderate correlations among DVSS dimensions parallel Zhang et al.’s conceptualization of digital validation seeking as manifesting through identity, competence, and social channels. Furthermore, the scale’s capacity to differentiate between compulsive feedback monitoring, performative presentation, comparative evaluation, and metric-based self-assessment addresses Nguyen and Habók’s call for instruments measuring why and how students seek validation online rather than merely assessing what they can do technologically, positioning the DVSS as a theoretically inalgrounded, context-sensitive tool.

The DVSS offers significant practical utility for higher education institutions seeking to identify students at risk for maladaptive digital engagement before patterns escalate to clinical impairment. Early identification of elevated validation-seeking behaviors enables targeted prevention programs addressing the psychological mechanisms underlying digital dependence, particularly among vulnerable populations including female students and those from lower socioeconomic backgrounds who experience heightened digital stressors. Educators can use DVSS profiles to design hybrid learning environments that deliberately channel validation drives toward collaborative learning and productive feedback rather than status comparison and fragmented attention. Platform architecture may differentially influence DVSS dimensions, with social media tools amplifying FHV and SC, while structured platforms like Blackboard may primarily engage ASQ. The scale’s multidimensional structure allows institutions to tailor interventions to specific behavioral patterns: students high in FHV may benefit from anxiety management strategies, while those scoring elevated on PS may require authentic engagement opportunities that reduce surface-level digital performance pressures and promote genuine academic involvement.

Several limitations warrant consideration when interpreting these findings. The cross-sectional design precludes causal inferences about relationships between digital validation seeking and academic or psychological outcomes. The Egyptian university sample, while geographically diverse, may not generalize to students in different cultural contexts where digital platform usage and validation-seeking behaviors differ substantially. Self-report methodology introduces potential social desirability bias, particularly for items addressing compulsive or performative behaviors. It should be noted that the psychometric properties of the DVSS were established exclusively within an Egyptian university context, and cross-cultural measurement invariance has not yet been empirically tested, precluding automatic extension of construct validity beyond this geographical scope. Additionally, the removal of one item (Q5) during factor analysis highlights the importance of iterative refinement in scale development. Finally, although demographic homogeneity between samples was confirmed across gender, academic year, and residence, unmeasured variables such as digital platform engagement intensity and socioeconomic status were not formally balanced, representing a remaining potential source of bias.

Future research should employ longitudinal designs tracking digital validation seeking across academic terms to examine temporal patterns, developmental changes, and prospective associations with academic performance, psychological well-being, and clinical outcomes such as burnout and mental health distress. Cross-cultural validation using multigroup CFA across diverse geographical and educational contexts represents a critical priority before broader application of the DVSS can be recommended. Researchers should investigate concurrent and predictive validity by examining correlations with established measures of academic engagement, SC orientation, SDT constructs, and mental health indicators. Experimental studies manipulating feedback availability or SC information could establish causal mechanisms underlying the virtual feedback loop. Finally, intervention research should evaluate whether DVSS-informed programs effectively reduce maladaptive validation seeking while preserving beneficial aspects of digital engagement for collaborative learning and academic support.

## 5. Conclusions

The DVSS represents a psychometrically sound, theoretically grounded instrument that fills a critical void in educational measurement by capturing the socio-emotional dimensions of digital behavior within higher education contexts. Its multidimensional structure reflects the complexity of contemporary student experiences navigating hybrid academic environments where digital platforms fundamentally shape learning, identity formation, and self-evaluation processes. By enabling early identification of maladaptive patterns before they compromise academic engagement or escalate to clinical thresholds, the DVSS equips researchers and practitioners with an essential tool for understanding and addressing the psychological implications of digitally mediated validation in university settings. As educational institutions increasingly integrate digital technologies into pedagogical practice, instruments like the DVSS become indispensable for ensuring that technological affordances enhance rather than undermine student well-being, authentic learning, and academic success.

## Figures and Tables

**Table 1:** Demographic Characteristics of Exploratory and CFA Samples.

Variable		Exploratory Sample (N = 511)		Confirmatory Sample (N = 740)	
		N	%	N	%
Gender	Male	271	53.0	389	52.6
	Female	240	47.0	351	47.4
Academic Year	First	127	24.9	181	24.5
	Second	125	24.5	188	25.4
	Third	140	27.4	184	24.9
	Fourth	119	23.3	187	25.3
Residence	Rural	243	47.6	335	45.3
	Urban	268	52.4	405	54.7
Platforms	Facebook	99	19.4	242	32.7
	TikTok	108	21.1	123	16.6
	Twitter	109	21.3	124	16.8
	Snapchat	104	20.4	124	16.8
	Instagram	91	17.8	127	17.1
University	Al-Azhar University	98	19.2	143	19.3
	Zagazig University	78	15.3	95	12.8
	Kafr El Shiekh University	74	14.5	140	18.9
	Beni Suef University	72	14.1	154	20.8
	Damanhour University	96	18.8	146	19.7
	Tanta University	93	18.2	62	8.5

**Table 2:** Descriptive Statistics for DVSS Items in Exploratory Sample.

Item	Mean	SD	Skewness	Kurtosis	K-S Statistic	K-S Sig.	S-W Statistic	S-W Sig.
Item1	3.10	1.41	−0.169	−1.234	0.154	0.000	0.885	0.000
Item2	2.57	1.30	0.213	−1.069	0.183	0.000	0.885	0.000
Item3	2.58	1.26	0.160	−1.041	0.167	0.000	0.893	0.000
Item4	2.49	1.28	0.278	−1.011	0.179	0.000	0.880	0.000
Item5	2.52	1.37	0.357	−1.115	0.195	0.000	0.867	0.000
Item6	2.58	1.36	0.231	−1.142	0.187	0.000	0.877	0.000
Item7	2.58	1.33	0.225	−1.115	0.177	0.000	0.882	0.000
Item8	2.43	1.30	0.355	−0.969	0.196	0.000	0.870	0.000
Item9	2.61	1.31	0.190	−1.178	0.177	0.000	0.888	0.000
Item10	2.57	1.34	0.265	−1.127	0.174	0.000	0.880	0.000
Item11	2.50	1.35	0.340	−1.109	0.183	0.000	0.870	0.000
Item12	2.44	1.30	0.349	−0.971	0.188	0.000	0.872	0.000
Item13	2.42	1.33	0.396	−1.045	0.197	0.000	0.860	0.000
Item14	2.50	1.39	0.359	−1.145	0.194	0.000	0.861	0.000
Item15	2.29	1.28	0.561	−0.819	0.218	0.000	0.847	0.000
Item16	2.64	1.29	0.264	−0.987	0.192	0.000	0.893	0.000
Item17	2.48	1.32	0.395	−1.020	0.189	0.000	0.872	0.000
Item18	2.39	1.31	0.444	−0.956	0.203	0.000	0.861	0.000
Item19	2.58	1.26	0.359	−0.878	0.191	0.000	0.892	0.000
Item20	2.41	1.34	0.461	−0.958	0.202	0.000	0.856	0.000

Note. K-S = Kolmogorov–Smirnov test (with Lilliefors significance correction); S-W = Shapiro–Wilk test. All Sig. values = 0.000 (*p* < 0.001), indicating significant departure from normality for all items.

**Table 3:** Total Variance Explained in EFA.

Factor	Initial Eigenvalues			Extraction Sums of Squared Loadings		
	Total	% of Variance	Cumulative %	Total	% of Variance	Cumulative %
1	9.490	47.452	47.452	9.072	45.360	45.360
2	1.590	7.952	55.404	1.201	6.007	51.366
3	1.105	5.525	60.930	0.690	3.448	54.815
4	0.933	4.663	65.593	0.496	2.480	57.294
5	0.714	3.568	69.161	–	–	–

**Table 4:** Parallel Analysis: Comparison of Real and Simulated Eigenvalues.

Factor	Real Data Eigenvalues	Simulated Data Mean Eigenvalues	Decision
1	10.140	0.455	Retain *
2	1.084	0.319	Retain *
3	0.595	0.266	Retain *
4	0.411	0.219	Retain *
5	0.111	0.189	–

Note. * = Factor should be retained based on real eigenvalue exceeding simulated eigenvalue.

**Table 5:** Pattern Matrix for Four-Factor Solution with Promax Rotation.

Item	Factor 1 (FHV)	Factor 2 (PS)	Factor 3 (SC)	Factor 4 (ASQ)	Uniqueness
Item6	0.718				0.358
Item7	0.664				0.397
Item8	0.702				0.369
Item9	0.812				0.322
Item10	0.901				0.217
Item16		0.611			0.435
Item17		0.720			0.245
Item18		0.748			0.275
Item19		0.793			0.322
Item20		0.740			0.319
Item11			0.719		0.402
Item12			0.661		0.354
Item13			0.854		0.281
Item14			0.684		0.311
Item15			0.571		0.371
Item1				0.690	0.417
Item2				0.809	0.295
Item3				0.506	0.540
Item4				0.605	0.389
Item5					0.581

Note. FHV = Feedback Hyper-vigilance; PS = Performative Studiousness; SC = Social Comparison; ASQ = Academic Self-Quantification. Loadings < 0.40 are suppressed for clarity.

**Table 6:** Factor Correlation Matrix for Four-Factor Solution.

Factor	1	2	3	4
1. FHV	1			
2. PS	0.620	1		
3. SC	0.625	0.736	1	
4. ASQ	0.655	0.744	0.683	1

**Table 7:** Fit Indices for Competing CFA Models.

Model	χ^2^	df	χ^2^/df	RMSEA	CFI	NFI	GFI	AGFI	RMR
Unidimensional	1693.676	170	9.963	0.110	0.812	0.795	0.762	0.707	0.119
First-Order Four-Factor	378.678	146	2.594	0.046	0.970	0.952	0.949	0.934	0.053
Second-Order	396.683	148	2.680	0.048	0.968	0.950	0.947	0.932	0.058

Note. Normed Fit Index (NFI), Goodness of Fit Index (GFI), Adjusted Goodness of Fit Index (AGFI), Root Mean Square Residual (RMR).

**Table 8:** Reliability Coefficients for DVSS Dimensions and Total Scale.

Dimension	Coefficient ω	Coefficient α	Guttman’s λ2	CR	AVE
ASQ	0.810	0.807	0.809	0.811	0.519
FHV	0.874	0.873	0.874	0.874	0.582
SC	0.871	0.871	0.871	0.871	0.574
PS	0.883	0.882	0.882	0.883	0.602
Total Scale	0.938	0.938	0.939	–	–

Note. CR = Composite Reliability; AVE = Average Variance Extracted.

**Table 9:** Intercorrelations Among DVSS Subscales and Total Score.

Dimension	ASQ	FHV	SC	PS	Total
ASQ	1				
FHV	0.612 **	1			
SC	0.610 **	0.570 **	1		
PS	0.660 **	0.571 **	0.697 **	1	
Total Score	0.832 **	0.820 **	0.858 **	0.870 **	1

Note. ** *p* < 0.01 (two-tailed).

## Data Availability

The datasets generated and analyzed during the current study are available from the corresponding authors upon reasonable request.

## Abbreviations

The following abbreviations are used in this manuscript:

DVSS	Digital Validation Seeking Scale
SDT	Self-Determination Theory
CVI	Content Validity Index
EFA	Exploratory Factor Analysis
CFA	Confirmatory Factor Analysis
KMO	Kaiser-Meyer-Olkin
RMSEA	Root Mean Square Error of Approximation
CFI	Comparative Fit Index
AGFI	Adjusted Goodness of Fit Index
RMR	Root Mean Square Residual
CR	Composite Reliability
AVE	Average Variance Extracted
ASQ	Academic Self-Quantification
FHV	Feedback Hyper-vigilance
SC	Social Comparison
PS	Performative Studiousness
GPA	Grade Point Average

## Appendix A. Digital Validation Seeking Scale (DVSS)

Response Instructions: Please read each statement carefully and select the response that best describes your feelings and behaviors. There are no right or wrong answers. Please be as honest as possible.

Likert Scale: 5 = Strongly Agree | 4 = Agree | 3 = Neutral | 2 = Disagree | 1 = Strongly Disagree

**Factor 1: Academic Self-Quantification (ASQ)**

1. I feel smarter when my academic posts receive high engagement.
2. I consider the number of “likes” as evidence of my academic success.
3. My academic confidence increases when others praise my digital achievements.
4. I feel embarrassed if my study-related updates do not get enough interaction.

**Factor 2: Feedback Hyper-vigilance (FHV)**

5. I check my phone constantly immediately after posting a study update.
6. I feel anxious if responses to my academic posts are delayed.
7. Waiting for notifications distracts me while I am studying.
8. I find it difficult to focus until I see how people have reacted to my posts.
9. I refresh my profile repeatedly to track the increase in interactions.

**Factor 3: Social Comparison (SC)**

10. I compare the engagement on my posts with those of my classmates.
11. I feel discouraged if a classmate’s post receives more likes than mine.
12. I monitor my peers’ digital success to evaluate my own academic standing.
13. I strive to exceed the “likes” received by the top students in my class.
14. I am curious to see which of my academic rivals viewed my posts.

**Factor 4: Performative Studiousness (PS)**

15. I focus more on photographing my books and tools than actually reading them.
16. I spend a lot of time “staging” my study space before I actually start.
17. I am mostly interested in appearing serious about my studies to my followers.
18. I post images that imply hard work even when I am not actually studying.
19. I choose my study locations based on how “Instagrammable” they are.

**Scoring:**

– Academic Self-Quantification (ASQ): Sum of items 1, 2, 3, 4 (Range: 4–20)
– Feedback Hyper-vigilance (FHV): Sum of items 6, 7, 8, 9, 10 (Range: 5–25)
– Social Comparison (SC): Sum of items 11, 12, 13, 14, 15 (Range: 5–25)
– Performative Studiousness (PS): Sum of items 16, 17, 18, 19, 20 (Range: 5–25)
– Total DVSS Score: Sum of all 19 items (Range: 19–95)
